# Biomechanical Effect of Margin Convergence Techniques: Quantitative Assessment of Supraspinatus Muscle Stiffness

**DOI:** 10.1371/journal.pone.0162110

**Published:** 2016-09-01

**Authors:** Taku Hatta, Hugo Giambini, Chunfeng Zhao, John W. Sperling, Scott P. Steinmann, Eiji Itoi, Kai-Nan An

**Affiliations:** 1 Biomechanics laboratory, Division of Orthopedic Research, Mayo clinic, Rochester, Minnesota, United States of America; 2 Department of Orthopedic Surgery, Mayo clinic, Rochester, Minnesota, United States of America; 3 Department of Orthopaedic Surgery, Tohoku university school of medicine, Sendai, Japan; Queen Mary University of London, UNITED KINGDOM

## Abstract

Although the margin convergence (MC) technique has been recognized as an option for rotator cuff repair, little is known about the biomechanical effect on repaired rotator cuff muscle, especially after supplemented footprint repair. The purpose of this study was to assess the passive stiffness changes of the supraspinatus (SSP) muscle after MC techniques using shear wave elastography (SWE). A 30 × 40-mm U-shaped rotator cuff tear was created in 8 cadaveric shoulders. Each specimen was repaired with 6 types of MC technique (1-, 2-, 3-suture MC with/without footprint repair, in a random order) at 30° glenohumeral abduction. Passive stiffness of four anatomical regions in the SSP muscle was measured based on an established SWE method. Data were obtained from the SSP muscle at 0° abduction under 8 different conditions: intact (before making a tear), torn, and postoperative conditions with 6 techniques. MC techniques using 1-, or 2-suture combined with footprint repair showed significantly higher stiffness values than the intact condition. Passive stiffness of the SSP muscle was highest after a 1-suture MC with footprint repair for all regions when compared among all repair procedures. There was no significant difference between the intact condition and a 3-suture MC with footprint repair. MC techniques with single stitch and subsequent footprint repair may have adverse effects on muscle properties and tensile loading on repair, increasing the risk of retear of repairs. Adding more MC stitches could reverse these adverse effects.

## Introduction

Rotator cuff tear is a common cause of shoulder pain and dysfunction, and its occurrence is increasing due to the aging population [1, 2]. The size of full-thickness rotator cuff tears often increases over time and symptomatic patients who failed conservative treatment may require surgeries to improve their shoulder function and/or decrease their pain [3–6]. Arthroscopic rotator cuff repair, to reconstruct the tendon-bone interface, has been a well-established surgical option with advanced technique and devices. However, treatment of those patients with large or massive rotator cuff tears still remains a challenge, with a reported prevalence from 20 to 70% of patients undergoing repair for large size tears presenting with a re-tear before intact healing is observed [7–9]. Especially, traditional footprint repair techniques in patients with longitudinal-type tears (*e*.*g*. U-shaped, L-shaped tear) exhibiting a long and narrow pattern with increased medial-lateral length of the tear, have been cautioned to cause significant strain at the repair site, with high prevalence of failure [10, 11].

Burkhart et al. [12] advocated an alternative “margin convergence (MC)” technique with an initial side-to-side repair for large size tears. This technique was suggested to prevent the apex of the tear from excessive tension and to provide an advantageous environment for intact healing in the post-repaired tendon [12–14]. In addition to clinical reports with satisfactory outcomes [15, 16], one biomechanical study previously investigated the effect of MC techniques on strain changes in rotator cuff tendons [17]. On the other hand, there have been no biomechanical studies evaluating the effect of the MC techniques on the muscular properties in repaired rotator cuff structures.

Shear wave elastography (SWE), an ultrasound technique, has been a recent focus for quantifying mechanical properties of soft tissues by measuring shear wave propagation speed. This has been successfully used clinically for breast cancers diagnosis [18] and liver fibrosis staging [19]. In addition, passive stiffness of skeletal muscles, including the supraspinatus (SSP) muscle, has been assessed using SWE by *in-vivo* and *ex-vivo* studies [20–24]. In the clinical setting, larger rotator cuff tears are frequently associated with chronic changes in rotator cuff muscles, including hypotrophy or degenerations. Previous studies regarding rotator cuff repair techniques have mostly focused on the properties of repaired tendon-bone interfaces [25–27]. However, biomechanical assessment of rotator cuff muscle properties should also be addressed to determine the advantage/disadvantage of each repair technique and the possible effects of each in the healing process and/or postoperative rotator cuff function.

The purpose of the current study was twofold: 1) to assess the mechanical properties of the SSP muscle after MC techniques; 2) to assess the variability in results based on single or multiple sutures for MC technique with/without footprint repair.

## Materials and Methods

### Specimen Preparation

Eight fresh-frozen intact shoulders from 8 human cadavers were obtained from the Mayo Clinic Anatomy Department after institutional review board approval from the Mayo Bio-specimens Sub-committee. Written informed consent was obtained from the family before the start of this research. Exclusion criteria included the presence of glenohumeral arthritic changes, rotator cuff tear, and prior shoulder surgeries. Before the experimental procedures, the scapulae were dissociated from the thorax, and the humerus was cut at the level of the midshaft. The scapula and a fiberglass rod inserted into the humeral medullary canal were attached to a custom-designed experimental fixture. According to the International Society of Biomechanics (ISB) recommendation, the scapula was secured at 0° of upward/downward rotation, considered as a neutral position [28, 29]. The fixture, designed to provide 6 degrees-of-freedom motion of the glenohumeral joint in consistent motion paths, was used to abduct the humerus parallel to the scapular plane.

### Rotator cuff tear and repair designs

**Fig 1:**
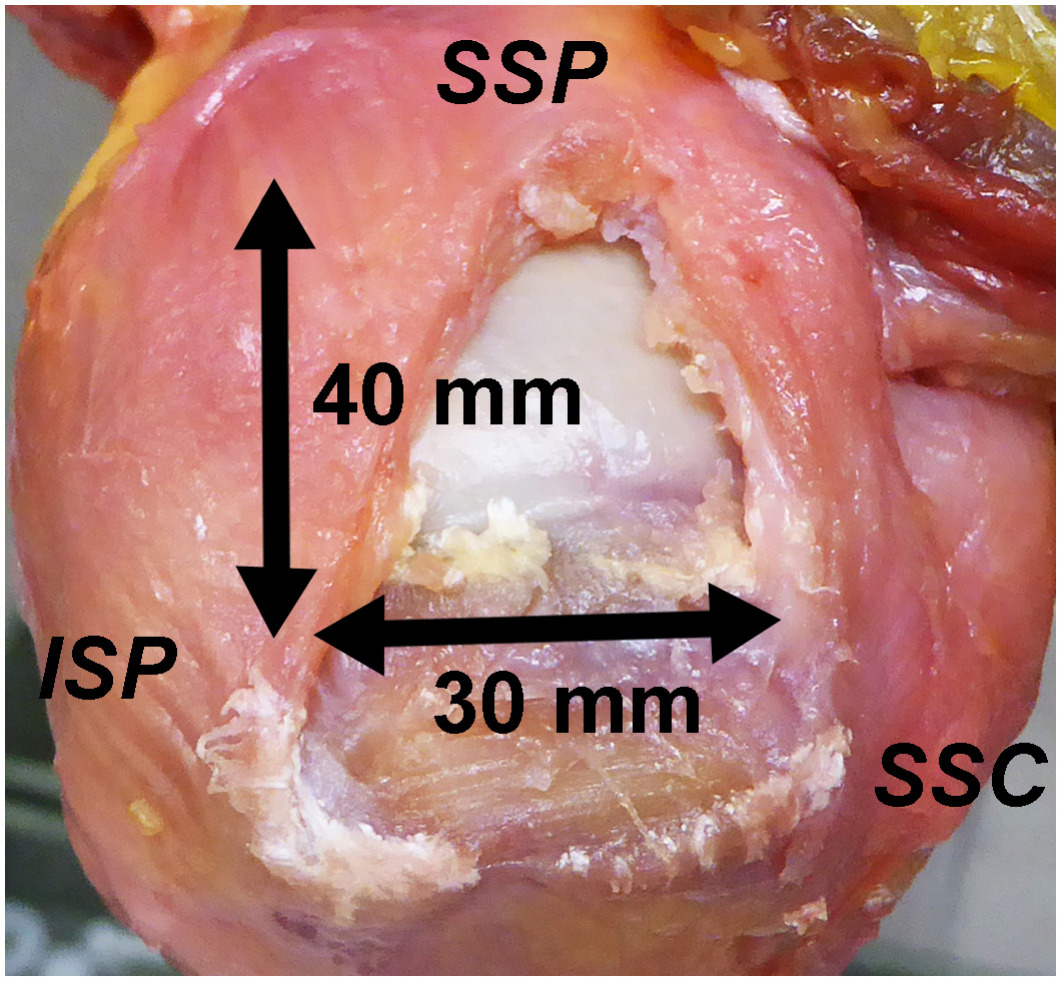
A large U-shaped rotator cuff tear was artificially created by removing rotator cuff tendon with 30 mm-width and 40 mm-length.

**Fig 2:**
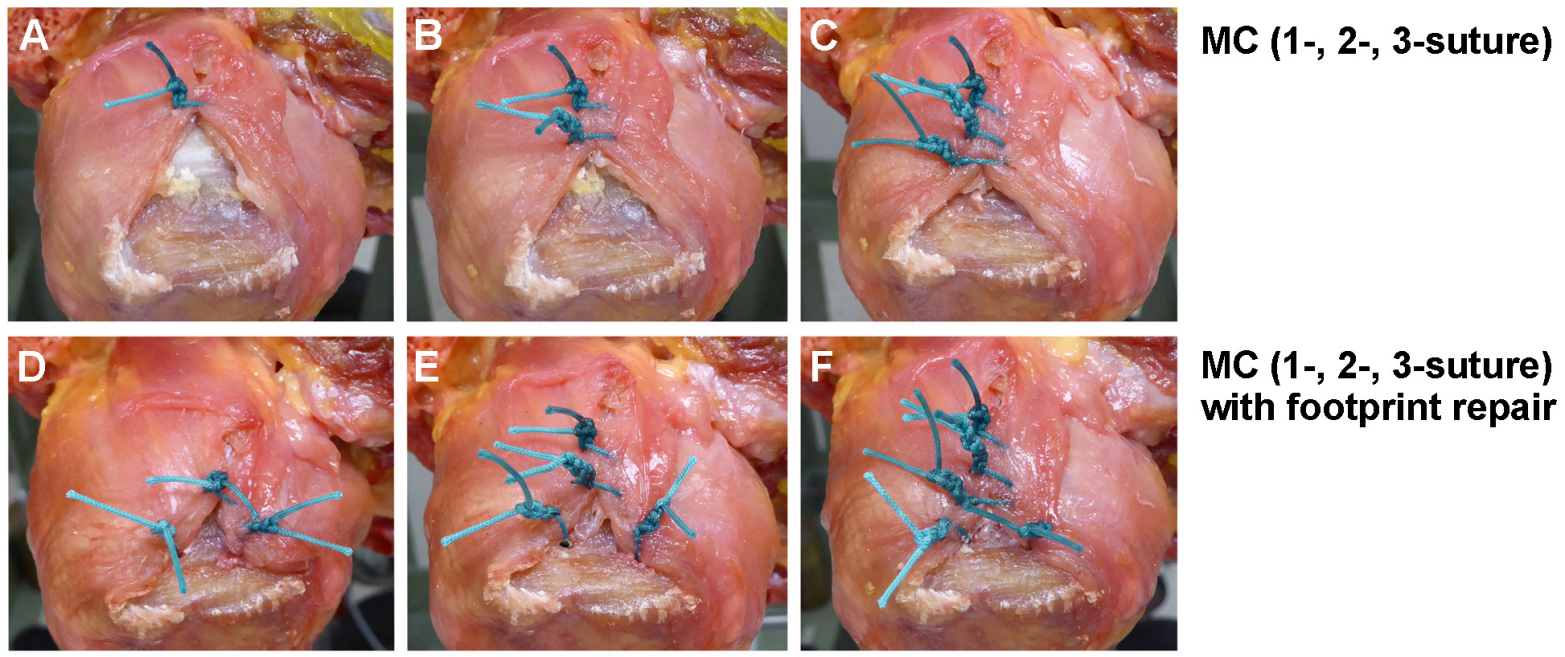
Six types of margin convergence (MC) technique. The MC was performed using one, two, or all three sutures (1-, 2-, 3-suture MC; A, B, C). Footprint repair was performed after MC techniques (1-, 2-, 3-suture MC with footprint repair; D, E, F).

A large U-shaped rotator cuff tear of 30 mm-width (anterior-posterior dimension) and 40 mm-length (medial-lateral dimension) was created in each shoulder, by removing tendinous tissue originating from the anterior margin of the SSP tendon and extending posteriorly along the greater tuberosity (Fig 1). Each tear was repaired using 6 types of MC techniques, with the shoulder position at 30° abduction and null rotation. Repair types were chosen in a random order for each specimen. Briefly, simple sutures for the MC technique were placed 10 mm, 20 mm, and 30 mm apart from the medial edge of the tendon. Thus, MC was performed using one, two, or all three sutures (1-, 2- or 3-suture MC, Fig 2A, 2B and 2C). In addition to these three MC techniques, two suture anchors (SwiveLock; Arthrex, Naples, FL) for footprint repair were placed at the level of the original footprint, 10 and 20 mm posterior to the bicipital groove. After these three types of MC techniques were completed, single-row footprint repair was performed to restore the footprint area with no gap, as previously described [15, 16] (1-, 2-, or 3-suture MC with footprint repairs; Fig 2D, 2E and 2F). In this study, one limb of the suture pairs from each anchor was passed though the tendon at the midpoint of both sides of the triangular gap originating after the MC suture process, and tied to the other end of the suture strand. A No. 2 Ethibond (Ethicon Inc., Somerville, NJ) was used for all sutures.

### Shear Wave Elastography

**Fig 3:**
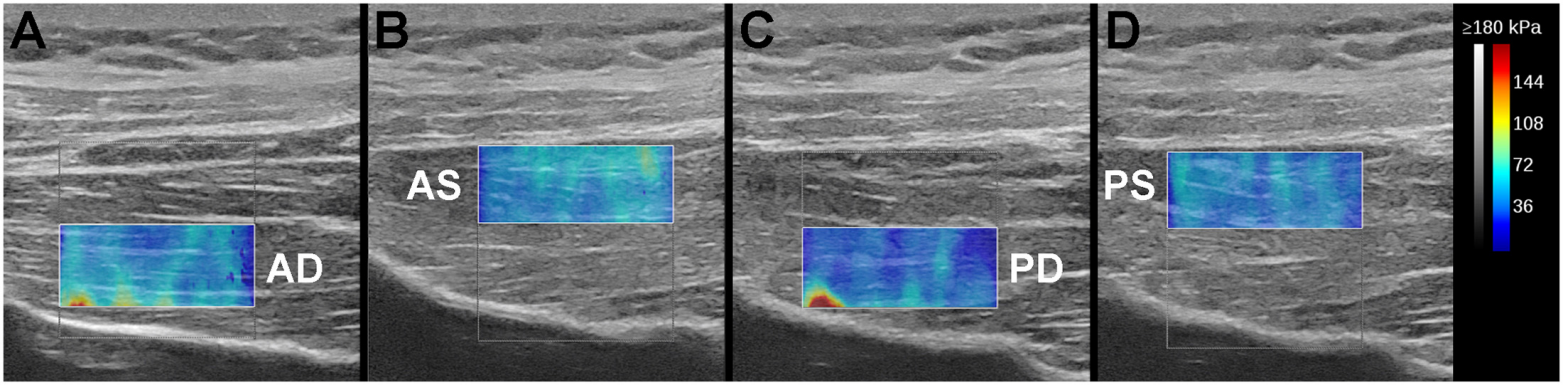
Quadrisected regions of the supraspinatus (SSP) muscle for shear wave elastography (SWE) measurements. The SSP muscles were divided into 4 regions: anterior deep (AD; A), anterior superficial (AS; B), posterior deep (PD; C), and posterior superficial (PS; D) based on the muscle fiber orientation.

For quantitative assessment of the SSP muscles stiffness, a commercial ultrasound system (Aixplorer; Supersonic Imagine, Aix-en-provence, France) with a 10–2 MHz transducer was used. Passive stiffness of the SSP muscle was measured based on an established SWE methodology [23, 24]. Briefly, the SSP muscle was divided into 4 regions according to the muscle fiber orientation; anterior deep (AD), anterior superficial (AS), posterior deep (PD), and posterior superficial (PS), as shown in Fig 3. SWE measurements for each region were assessed independently on a plane parallel to the muscle fibers. All SWE experiments were performed with the shoulder position at 0° abduction and null rotation, in order to assess the change in muscular properties under tensile stress [23]. We compared SWE values obtained under 8 different conditions: *intact* (before making a tear), *torn*, and *postoperative conditions* with 6 techniques.

The ratio of increased muscle stiffness for each repair was calculated as follows:

$$\frac{SWE\_values\_after\_repair}{SWE\_values\_in\_\text{int}act\_condition}x100(\%)$$

The values for the four regions (AD, AS, PD, and PS) were compared to assess regional stiffness variability within the SSP muscles.

### Statistical Analyses

Friedman with Dunn’s post hoc test was used to compare SWE values among the 8 conditions (intact, torn, and after repair with 6 techniques). This test was also used to assess the difference in increased ratio among the 4 muscular regions (AD, AS, PD, and PS) after each repair condition. These non-parametric tests were adopted since our data did not present a normal distribution. The specimen number used in this study is similar to or more than other biomechanical studies investigating repair techniques [25, 30, 31]. The significance level was set to P < 0.05.

## Results

**Fig 4:**
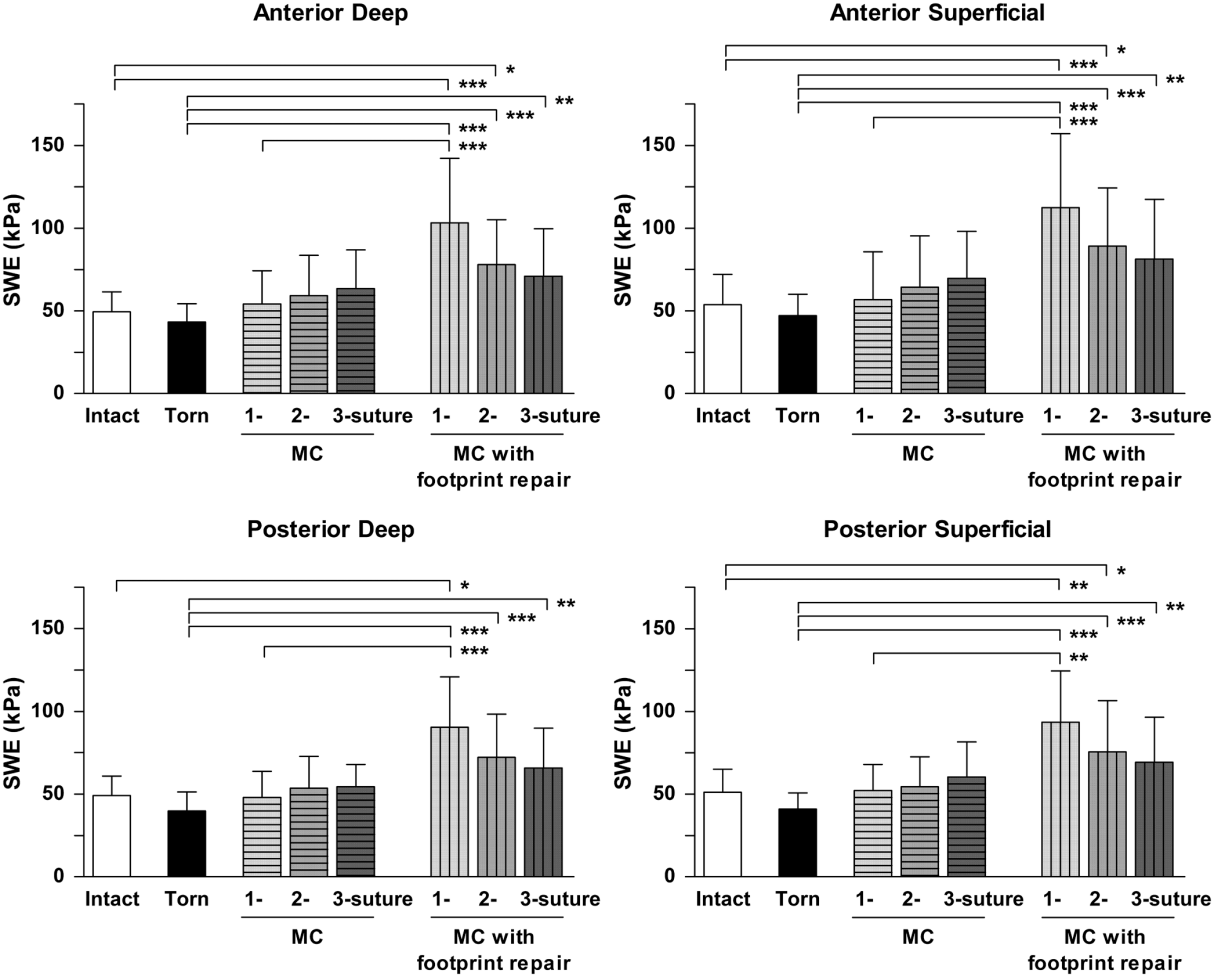
Alteration of SWE values in four muscular regions after MC techniques with/without footprint repair. *: P < 0.05, **: P < 0.01, ***: P < 0.001.

The mean (SD) SWE values of the intact SSP muscles were 49.6 kPa (SD, 12.0) for AD, 53.7 kPa (SD, 18.4) for AS, 49.1 kPa (SD, 11.7) for PD, and 51.1 kPa (SD, 14.1) for the PS region (Fig 4). After the artificial tear was created, SWE values showed an expected decrease in the values for all regions. Rotator cuff repair using all six techniques was successfully performed in all specimens. MC techniques using 1-, 2-, or 3-suture combined with footprint repair showed higher SWE values than unrepaired torn condition (for all 4 regions). Moreover, compared to the intact cuff condition, SWE values were significantly increased after footprint repairs with initial MC techniques using 1-suture (for all 4 regions) and 2-suture (for AD, AS, and PS). There was no significant difference between the intact condition and a 3-suture MC with footprint repair. Although not statistically significant, SWE values of the SSP muscle after a 1-suture MC with footprint repair were higher for all regions when compared among all procedures with multiple MC repairs.

**Fig 5:**
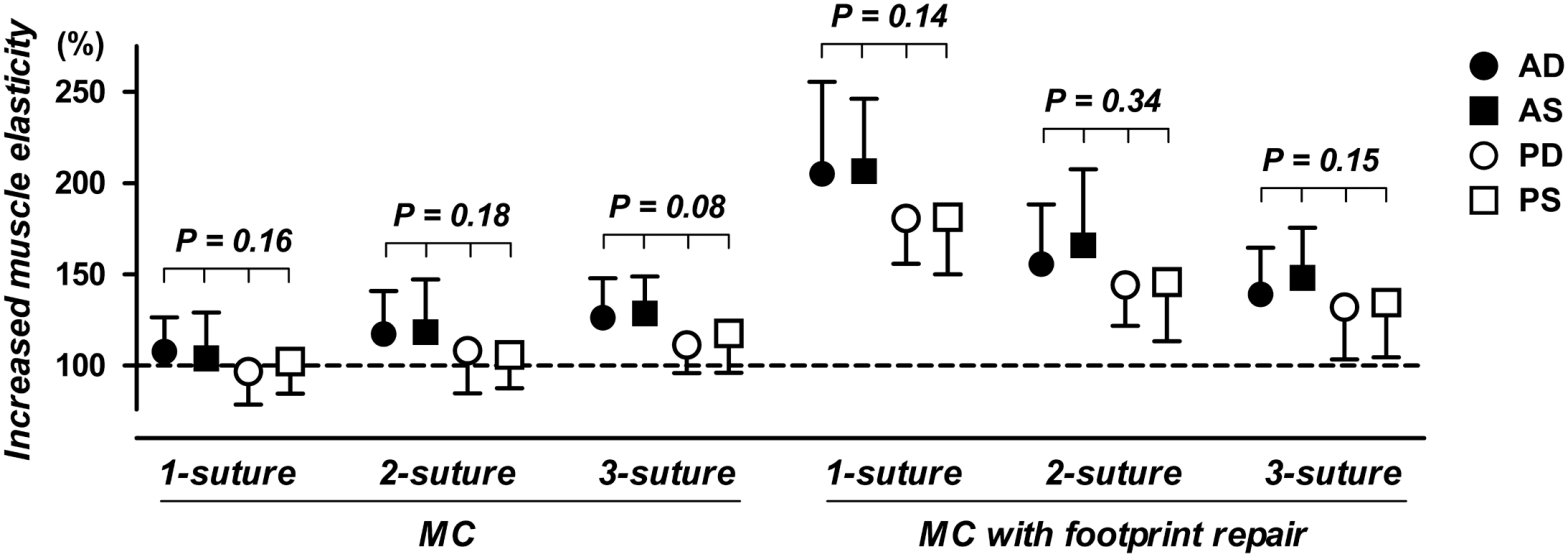
Increased SSP muscle stiffness after repairs using 6 MC techniques. Each dot represents the mean ratios of increased stiffness with the shoulder position at 0° abduction after repairs (error bars represent standard deviations). Preoperative (intact) values of the SSP muscle stiffness were set as 100% (dotted line).

The ratio of increased muscular stiffness after repairs showed no significant differences among the four regions regardless the type of repair techniques. However, shoulders with 3-suture MC technique (P = 0.08), and 1-suture MC with foot print repairs (P = 0.14) presented a higher trend in SWE values in the anterior regions after repair when compared to the posterior muscular regions (Fig 5).

## Discussion

This study investigated the biomechanical effect of margin convergence (MC) techniques on a rotator cuff muscle using a quantitative elastographic method. Our results demonstrated patterns of passive stiffness changes in the SSP muscle which underwent single or advanced, multiple MC techniques with and without subsequent footprint repair. Notably, when MC technique was solely performed, the number of MC sutures showed minimal variation of the SSP muscle stiffness. If a footprint repair was added to the MC, however, muscular properties based on SWE measurements showed altered outcomes according to the type of MC techniques performed. Among the 6 types of repair methods, 1-suture MC followed by footprint repair showed the highest SWE values in the SSP muscle. In contrast, multiple MC sutures with footprint repair could suppress the excessive stiffness in the SSP muscle due to a shifting of the free margin of the tendon edge toward the greater tuberosity, thus reducing the stresses for the subsequent footprint repair. Because enlargement of a tear size is biomechanically caused by an increased strain at the tendon edge, this regulated mechanical environment in rotator cuff muscle is important for repaired rotator cuff tendons during the healing process [32, 33], specially immediate to the postoperative period before tendon healing occurs. In addition, passive stiffness changes in the rotator cuff muscles after repair might directly correspond to postoperative recovery on rotator cuff function.

SWE has been used to quantify the mechanical properties of various skeletal muscles [20, 21, 34]. Due to the relatively complicated fiber architectures of the SSP muscle, the implementation of this technique required an independent segmental assessment of quadrisected muscular regions, which has been advocated to be reliable and feasible [23, 24, 35]. Furthermore, this technique has the capability to detect changes in mechanical properties based on varied passive tensions due to shoulder abduction-adduction in cadaveric shoulders [23]. Based on these features, therefore, we assessed a maximal passive stiffness, assumed to be seen in an adducted shoulder position with maximal elongation of the SSP muscle.

This study reinforces the advantage of MC techniques with a novel an insight in rotator cuff muscle properties. Burkhart [36] classified rotator cuff tears based on shapes and mobility (crescent, U-shaped, L-shaped, and massive). Additional studies have cautioned about footprint repairs for large U-shape tear, since securing the tendon-bone with suture anchors only may result in a high tension at the tendon-bone interface with higher rates of failure [10, 11]. However, if the free tendon edge is repaired to the bony footprint after MC, less tension at the tendon-bone interface could be observed compared to repairing directly onto the footprint without MC [12–14]. To our knowledge, only one study has previously investigated the biomechanical effects of MC techniques. The authors developed an SSP-deficient cadaveric shoulder as a massive tear model and demonstrated that the MC technique could decrease the strain in the residual rotator cuff tendon (subscapularis and infraspinatus) as well as the gap size of the defect [17]. Our results also support biomechanical features that MC techniques with more sutures could suppress excessive passive stiffness of muscular regions in the rotator cuff.

Mazzocca et al. described in an artificial massive-tear cadaveric study a detailed analysis of MC techniques, such as the number or location of MC sutures [17]. In their study, the authors performed 5 types of MC techniques with 5 simple sutures placed 5 mm apart starting medially at the glenoid rim and proceeding laterally. They showed the first, medial MC suture, was able to largely decrease the gap size (50% gap closure with first suture), and reduce the strain in both the subscapularis and infraspinatus tendon throughout the rotation angles in an adducted shoulder position. When increasing the number of MC sutures, the authors found a decrease gap size but less dramatic compared the first, medial MC suture. In particular, there were no progressive effects on strain suppression with multiple MC sutures. Although their study focused on the simple effect of MC techniques without any footprint repair, the findings obtained from a massive rotator cuff tear model provided important information relating MC techniques.

In contrast, the present study addressed 6 types of MC techniques, matching those observed in the clinical setting. To date, several clinical reports have shown satisfactory outcomes of arthroscopic MC repair in association with subsequent fixation onto the bony footprint [15, 16] or without footprint repair [37]. Focusing on the MC techniques with/without subsequent footprint repair, we first evaluated their combined effects on the mechanical properties in the SSP muscle. Accordingly, our results indicated that a single MC technique to increased muscle stiffness after footprint repair. Thus, multiple MC technique using various side-to-side sutures might be preferred if subsequent footprint repair is indicated.

There are several limitations in this study. First, our results were obtained from cadavers. Despite our results providing information only at time 0, these findings offer significant evidence relating the period immediately after surgery. Although *in vivo* SWE measurements might present variability based on time of measurement acquisition (from repair to examination), future studies using *in vivo* subjects would significantly complement our results. Second, rotator cuff tears were created by removing the normal tendons for the assessment of repair. Although this method of tear creation has been used in a number of biomechanical studies, we should note this may not duplicate the degenerative nature of chronic tears *in vivo*. Third, we applied a standardized large U-shaped tear model for the assessment of MC techniques. Although the location and size of the articular tear is considered a common pattern similar to a large tear, muscular conditions after repair might have differed among the positions of original tear. In addition to the SSP muscle, future studies should also investigate other rotator cuff muscle structures such as the subscapularis and infraspinatus. Fourth, standardization of the tension on the muscle-tendon was performed with arm position rather than with a tensioning device. When positioning the arm at 0°, the muscle-tendon will undergo stretching with an increase in stiffness values measured by SWE [23]. However, we believe that this approach is adequate as it allows for the muscle-tendon complex structure to be repaired implementing clinical considerations, while allowing for measurements to be obtained at a constant neutral position in all cadaver specimens. Finally, we did not measure the repair strength of the six different repair techniques.

## Conclusions

In conclusion, we have demonstrated the biomechanical effects on the rotator cuff muscle due to varying MC techniques. Our results revealed that single stitch MC repair combined with footprint repairs could increase muscle stiffness, thus increasing the tensile loading on the repair site. By adding multiple MC sutures, we could reverse these adverse effects of footprint repairs.
